# Identifying Mild Alzheimer's Disease With First 30-Min ^11^C-PiB PET Scan

**DOI:** 10.3389/fnagi.2022.785495

**Published:** 2022-04-05

**Authors:** Chushu Shen, Zhenguo Wang, Hongzhao Chen, Yan Bai, Xiaochen Li, Dong Liang, Xin Liu, Hairong Zheng, Meiyun Wang, Yongfeng Yang, Haifeng Wang, Tao Sun

**Affiliations:** ^1^Paul C. Lauterbur Research Center for Biomedical Imaging, Shenzhen Institute of Advanced Technology, Chinese Academy of Science, Shenzhen, China; ^2^Henan Provincial People's Hospital and the People's Hospital of Zhengzhou, University of Zhengzhou, Zhengzhou, China

**Keywords:** Alzheimer's disease, ^11^C-PiB PET, β-amyloid, imaging protocol, dynamic imaging

## Abstract

**Introduction:**

^11^C-labeled Pittsburgh compound B (^11^C-PiB) PET imaging can provide information for the diagnosis of Alzheimer's disease (AD) by quantifying the binding of PiB to β-amyloid deposition in the brain. Quantification index, such as standardized uptake value ratio (SUVR) and distribution volume ratio (DVR), has been exploited to effectively distinguish between healthy and subjects with AD. However, these measures require a long wait/scan time, as well as the selection of an optimal reference region. In this study, we propose an alternate measure named amyloid quantification index (AQI), which can be obtained with the first 30-min scan without the selection of the reference region.

**Methods:**

^11^C-labeled Pittsburgh compound B PET scan data were obtained from the public dataset “OASIS-3”. A total of 60 mild subjects with AD and 60 healthy controls were included, with 50 used for training and 10 used for testing in each group. The proposed measure AQI combines information of clearance rate and mid-phase PIB retention in featured brain regions from the first 30-min scan. For each subject in the training set, AQI, SUVR, and DVR were calculated and used for classification by the logistic regression classifier. The receiver operating characteristic (ROC) analysis was performed to evaluate the performance of these measures. Accuracy, sensitivity, and specificity were reported. The Kruskal–Wallis test and effect size were also performed and evaluated for all measures. Then, the performance of three measures was further validated on the testing set using the same method. The correlations between these measures and clinical MMSE and CDR-SOB scores were analyzed.

**Results:**

The Kruskal–Wallis test suggested that AQI, SUVR, and DVR can all differentiate between the healthy and subjects with mild AD (*p* < 0.001). For the training set, ROC analysis showed that AQI achieved the best classification performance with an accuracy rate of 0.93, higher than 0.88 for SUVR and 0.89 for DVR. The effect size of AQI, SUVR, and DVR were 2.35, 2.12, and 2.06, respectively, indicating that AQI was the most effective among these measures. For the testing set, all three measures achieved less superior performance, while AQI still performed the best with the highest accuracy of 0.85. Some false-negative cases with below-threshold SUVR and DVR values were correctly identified using AQI. All three measures showed significant and comparable correlations with clinical scores (*p* < 0.01).

**Conclusion:**

Amyloid quantification index combines early-phase kinetic information and a certain degree of β-amyloid deposition, and can provide a better differentiating performance using the data from the first 30-min dynamic scan. Moreover, it was shown that clinically indistinguishable AD cases regarding PiB retention potentially can be correctly identified.

## Introduction

Alzheimer's disease (AD) is an irreversible neurodegenerative disease that is characterized by dementia symptoms such as memory loss and cognitive impairment (Winblad et al., 2016). Currently, the diagnosis of AD is mainly based on clinical symptoms, while the presence of pathologically relevant biomarkers, including amyloid plaques and neurofibrillary tangles, could help to confirm the results and enable early detection (Jellinger, 1998). With radiotracers specific to β-amyloid plaques, PET imaging provides a useful tool for quantifying β-amyloid deposition in the brain regions. In 2019, the IDEAS (Imaging Dementia-Evidence for Amyloid Scanning) study involving 18,295 patients with mild cognitive impairment (MCI) or dementia and 946 dementia experts proved that implementing amyloid PET scanning would lead to higher diagnostic certainty, changing patient management and leading to improved outcomes (Rabinovici et al., 2019). ^11^C-labeled Pittsburgh compound B (PiB) is a radiotracer that performs *in vivo* imaging of amyloid deposition (Klunk et al., 2004). Previous studies suggested that a significant difference in PiB retention was observed in areas known to contain amyloid deposition, such as frontal, parietal cortex, and striatum (Klunk et al., 2004; Forsberg et al., 2010; Tryputsen et al., 2015). PiB PET imaging has been successfully used in discriminating AD, MCI, and healthy subjects (Lowe et al., 2009) as well as predicting MCI progression (Forsberg et al., 2008).

The most widely used quantification measures for ^11^C-PiB imaging are standardized uptake value ratio (SUVR) and distribution volume ratio (DVR). SUVR measures the ratio of SUV in target and reference regions over a late-scan period. The value of SUVR reflects the degree of PiB retention and thus the amyloid deposition in the region of interest (ROI) at the equilibrium stage of tracer distribution. This semi-quantitative method works effectively in assisting AD diagnosis, although it was known to suffer from non-specific tracer binding (Liu et al., 2021). DVR is the ratio of distribution volume from a receptor-containing region (target region) to a non-receptor region (reference region), which can be obtained by Reference Logan Graphical analysis (Logan et al., 1996). In PiB imaging, the DVR value reflects the equilibrium distribution of PiB and is significantly higher for subjects with AD in regions with β-amyloid deposition than normal. Apart from DVR and SUVR, relative tracer flow (R1) has also been reported to provide information for differentiating subjects with AD and HC (Peretti et al., 2019b; Ponto et al., 2019). It is defined as the ratio of tracer influx rate in the target region to that in the reference region, which measures the transport of tracer from plasma to tissue at the initial scan. Both DVR and R1 can be derived by fitting the simplified reference tissue model (SRTM) to the dynamic PET data (Lammertsma and Hume, 1996). Previous studies reported that R1 generated by the SRTM2 model is highly correlated with regional cerebral blood flow (Meyer et al., 2011) as well as FDG SUVR (Peretti et al., 2019c), and thus can serve as a biomarker of neuronal activity and neurodegeneration.

Although these measures have been proved useful for AD diagnosis, there are some issues with the current workflow. For example, the total scan and wait time for SUVR/DVR would add up to at least 1 h as they measure the tracer uptake at the late equilibrium state. While R1 can be estimated using early-stage PET data, it serves as a potential surrogate for FDG SUVR and is not directly correlated to amyloid quantification (Meyer et al., 2011; Peretti et al., 2019b). Moreover, all the three methods involve selecting a reference region without specific binding. The most frequently used reference region, the cerebellum, however, has been reported to have higher PiB retention in subjects with higher cortical β-amyloid deposition, which could in turn blur the significant results of β-amyloid deposition in target regions (Price et al., 2005).

In this study, we proposed an alternate measure for AD identification based on dynamic PiB PET data. The aim is to achieve comparable or even better discriminative performance on mild AD identification with a short scan time and not using the reference region for calculation. The proposed measure, amyloid quantification index (AQI), requires only the first 30-min scan which reflects both clearance rate from tissue at the early stage and PiB retention before equilibrium. Its performance in differentiating mild AD and HC subjects was assessed and compared with those of SUVR and DVR. Limitations and future work were discussed at the end of this paper.

## Materials and Methods

### Participants and Cognitive Assessments

A total of 60 mild AD subjects and 60 healthy controls (HCs) from the OASIS-3 dataset (LaMontagne et al., 2019) were included. AD scans were selected as those confirmed by two clinical diagnoses before and after the scan time. Both the clinical diagnoses for AD and non-AD dementia were made based on the National Alzheimer Coordinating Center Uniform Data Set (UDS) (Morris et al., 2006) assessments. Patients with non-AD dementia were excluded. Sixty-four PiB scans satisfied these criteria. Four scans were deserted due to the problem of missing necessary scan data. Among the remaining 60 scans, 50 were included in the training set and 10 were included in the testing set. HC scans were selected in the order of serial number, excluding subjects with AD and those with other diseases. In this study, AD_001 indicates the 1st AD subject while HC_001 indicates the 1st HC subject. Demographics of all subjects can be found in Table 1. Clinical and neuropsychological assessments were performed on all subjects prior to scans. Each subject received a clinical dementia rating (CDR) score, with a CDR of 0 indicating normal cognitive function and 0.5 or 1 indicating cognitive impairment. Confirmed subjects with AD were clinically diagnosed as “AD dementia”. As participants reaching CDR = 2 were no longer eligible for the study, here only mild and very mild AD cases were included (0.5 ≤ CDR ≤ 1 or 0.5 ≤ CDR-SOB ≤ 9) (LaMontagne et al., 2019). To obtain more accurate assessment results we use CDR-SOB (O'Bryant et al., 2008) to evaluate the degree of dementia for each subject, with the score being 0 for HCs and ranging from 0.5 to 9.0 for patients with AD (Sendi et al., 2021). General cognitive status was also evaluated for each subject through the Mini-Mental State Examination (MMSE), with scores ranging from 0 (severe impairment) to 30 (no impairment) (Tombaugh and McIntyre, 1992).

**Table 1:** Demographic information of 120 subjects by group.

	**AD group**	**HC group**
No. of subjects	60	60
Age (yr)	76.2 ± 7.2	66.5 ± 8.1
Sex (M/F)	45/15	22/38
ApoE 4 positive (%)	36/60	19/60
MMSE	25.7 ± 2.9	29.4 ± 1.0
CDR-SOB	3.2 ± 2.1	0

*MMSE, Mini-Mental State Examination; ApoE, apolipoprotein E; CDR-SOB, Clinical Dementia Rating Scale Sum of Boxes*.

### Imaging and Post-processing

^11^C-labeled Pittsburgh compound B (PiB) PET imaging was performed on each subject. Subjects were given 6–20 mCi ^11^C-labeled PiB intravenously. Dynamic scans (60 mins;12 x 10 s, 3 × 60 s, 11 × 5 min) were conducted on one of the three Siemens PET scanners: ECAT HR+ 962 PET, Biograph 40 PET/CT, and BioGraph mMR PET-MR. PET imaging analysis was performed as follows (LaMontagne et al., 2019). Reconstructed images were first smoothed to achieve a spatial resolution of 8 mm. Motion correction was applied to each set of dynamic images with an extensive frame-by-frame registration procedure. No partial volume or entropy corrections were applied. Brain parcellation was performed for each subject by registering PET images to the corresponding T1-weighted MR images, which had been segmented using FreeSurfer 5.3 (http://surfer.nmr.mgh.harvard.edu). Reference region-based Logan graphical analysis was implemented on each segmented region to calculate DVR (Logan et al., 1996). Regional SUVR was estimated for all the regions. Both DVR and SUVR used 30–60 min post-injection as the time window with the cerebellar cortex as the reference region.

### Use Short Scan Data

The first 30-min dynamic data in 100 subjects of the training set were used to exploit optimal features which can effectively distinguish between AD and HC subjects. The mean uptake over time for each brain region was quantified as time-activity curves (TACs). Linear interpolation was performed on TACs to obtain a fine sampling time for all scans.

**Figure 1:**
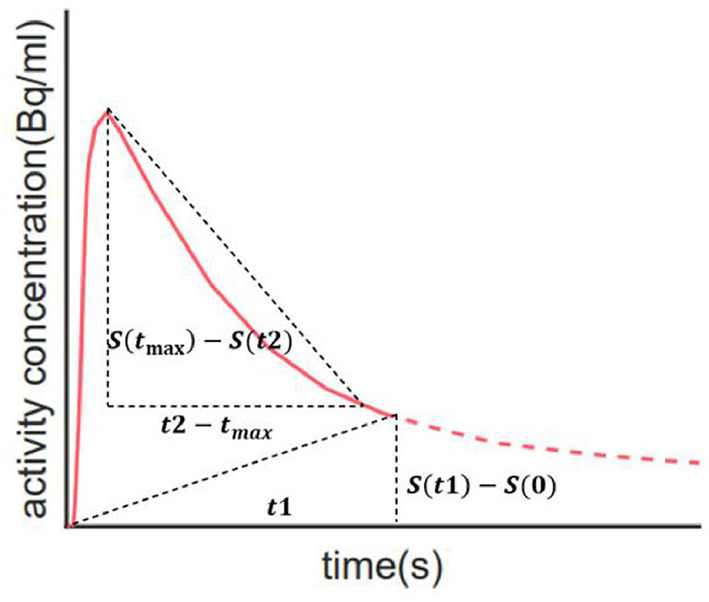
Illustration of how index AQI was calculated. The two oblique dashed lines represent the two slopes that reflect clearance rate and PiB retention respectively. Calculation of AQI only requires 30-min scan, as indicated by the solid red line.

According to the kinetics of PiB (Rodell et al., 2013), each TAC was split into three phases: flow-in phase, peak uptake, and clearance from tissue. The flow-in phase denotes the initial clearance of PiB by tissue, the rate of which is determined by cerebral blood flow and vascular permeability. The peak uptake phase describes the time when maximal tracer uptake was reached, generally within 4 min from the start (Gjedde et al., 2013). The clearance phase denotes the clearance of tracer from tissue after reaching the peak value, the rate of which can reflect amyloid load in the ROI. Compared with HCs, the AD group usually features greater PiB retention together with a lower clearance rate (Engler et al., 2006; Peretti et al., 2019a). Therefore, it is assumed that the combination of these two characteristics would work effectively in discriminating between diseased and healthy subjects. Based on this assumption, we proposed AQI. For each ROI, we calculated the descending slope from peak to a time point t1 afterward as well as the slope between the start point and a later time point t2 on the corresponding TAC (Figure 1). The first slope reflects the clearance rate whereas the second measures the PiB retention in mid-stage scans. Then these two slopes were linearly combined to yield the index AQI_roi in each ROI:


(1) $$\begin{array}{l} \text{AQI\_roi}=a\times \frac{S\left(t1\right)-S\left(0\right)}{t1}-\left(1-a\right)\times \frac{S\left(t_{\max}\right)-S\left(t2\right)}{t2-t_{\max}} \end{array}$$

Here *t* is the middle time point of each dynamic frame and t_max_ denotes the frame where peak uptake value occurs. S(t) represents the activity concentration (Bq/ml) of PiB as a function of t. S(0) is the average activity concentration of the first frame (0–10 s). Normalization was performed using injected dose for each scan. The optimal values for t1 and t2 and the coefficient “a” were determined by maximizing the classification accuracy. Then, a 10-fold cross-validation procedure using logistic regression classifier was applied to evaluate the performance of AQI_roi on differentiating subjects in the training set. The parameters that had the best compromise between accuracy and scan time were chosen to be the optimal value.

### Selection of Optimal Brain Regions

Conventional analysis of quantification methods is based on single or several regions known to contain amyloid deposition without investigating optimal regions. In this study, we identified featured brain regions for AQI using lasso regression analysis (Tibshirani, 1996). Lasso regression could perform variable selection as well as generalized linear regression by finding a set of coefficients β such that the sum of Mean Squared Error (MSE), and the regularization term can be minimized. Here the optimal regularization strength was empirically chosen as the largest value such that MSE is within one standard error of the minimum MSE. Predictors with relatively large coefficients were considered featured brain regions, the AQI_roi of which were linearly combined to distinguish between the AD and HC groups.

### Statistical Analysis

Statistical analysis was performed using MATLAB Statistics and Machine Learning Toolbox (version R2018b). The discriminatory performance of index AQI was compared with those of SUVR and DVR. Here the value of SUVR and DVR were calculated as the average values in anterior cingulate, frontal cortex, parietal cortex, and precuneus, which have been reported to accompany higher amyloid binding in subjects with AD than in HC (Klunk et al., 2004; Tryputsen et al., 2015). AQI was calculated linearly by combining the AQI_roi in featured brain regions, the coefficients of which were determined by linear regression. To test the performance of each measure, a 10-fold cross-validation was implemented by randomly partitioning the training subjects into 10 subsets, each containing five AD and five HC subjects. A logistic regression classifier was trained using nine subsets as training data and validated on the remaining subset. The process was repeated 10 times. Then, ROC analysis was performed to compare the classification results of these 10 iterations with true labels, and the sensitivity, specificity, accuracy, area under the curve (AUC), and optimal threshold were reported. To further validate the performance of the three methods, we used an additional 10 AD and 10 HC scans as the testing set. For each subject., AQI, SUVR, and DVR were calculated, as previously mentioned, for training sets. The logistic regression classifier that was trained with the previous 100 subjects was then applied to the testing set. Results of the ROC analysis and the above evaluation metrics were reported and compared. Moreover, the correlations between the three measures (SUVR/DVR/AQI) and clinical scores (MMSE/CDR-SOB) were analyzed using linear regression. The correlation coefficient and *p-*value were reported for each pair of variables.

## Results

### Summarized TACs in Sampled Regions for all Subjects

**Figure 2:**
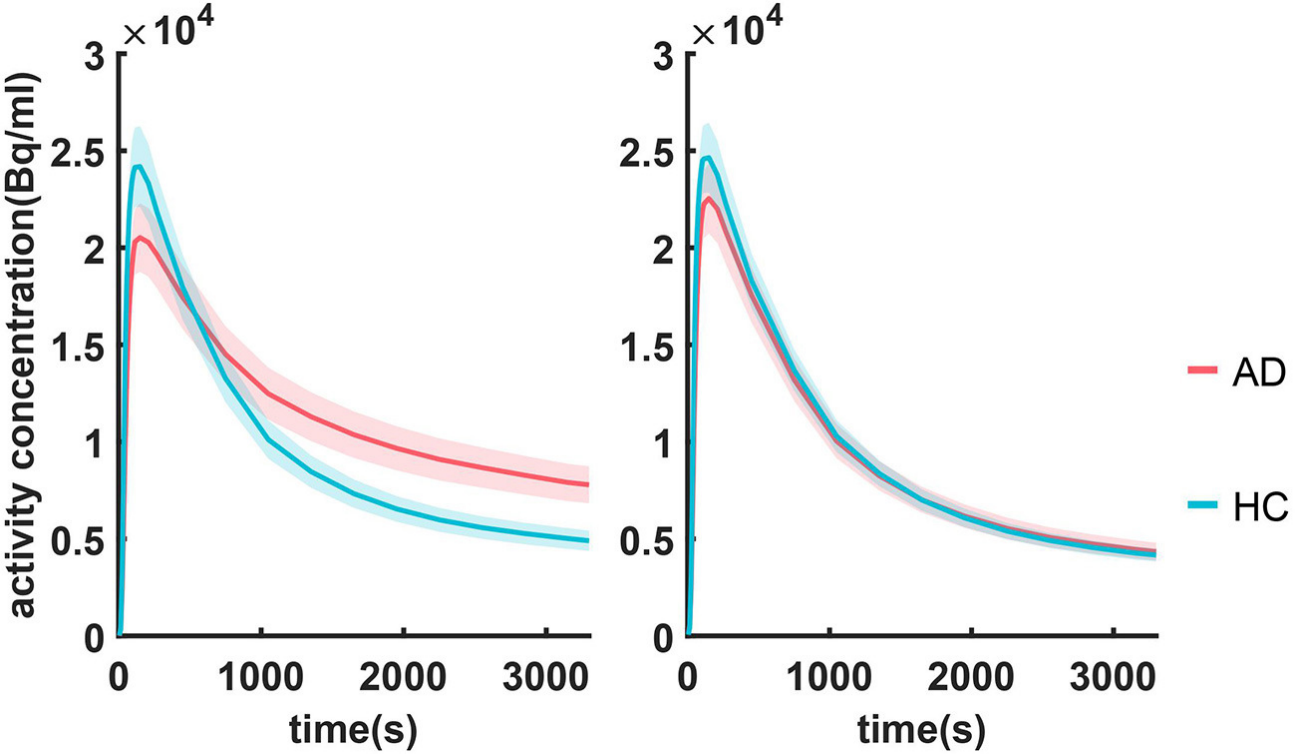
Summarized TACs for all 120 subjects in **(A)** caudal anterior cingulate cortex and **(B)** cerebellar cortex. The solid lines indicate that the mean value in each group and the shadowed areas indicate 95% confidence interval.

Summarized TACs for all 120 subjects in the caudal anterior cingulate cortex and cerebellar cortex are shown in Figure 2. Compared with HC, subjects with mild AD feature lower clearance rate and greater PiB retention in the caudal anterior cingulate cortex, whereas in the cerebellar cortex TACs for these two groups are similar due to the lack of specific binding. The difference in the dynamic uptake of certain brain regions allows AD and HC subjects to be separated.

### Optimal Parameters for Index AQI

**Figure 3:**
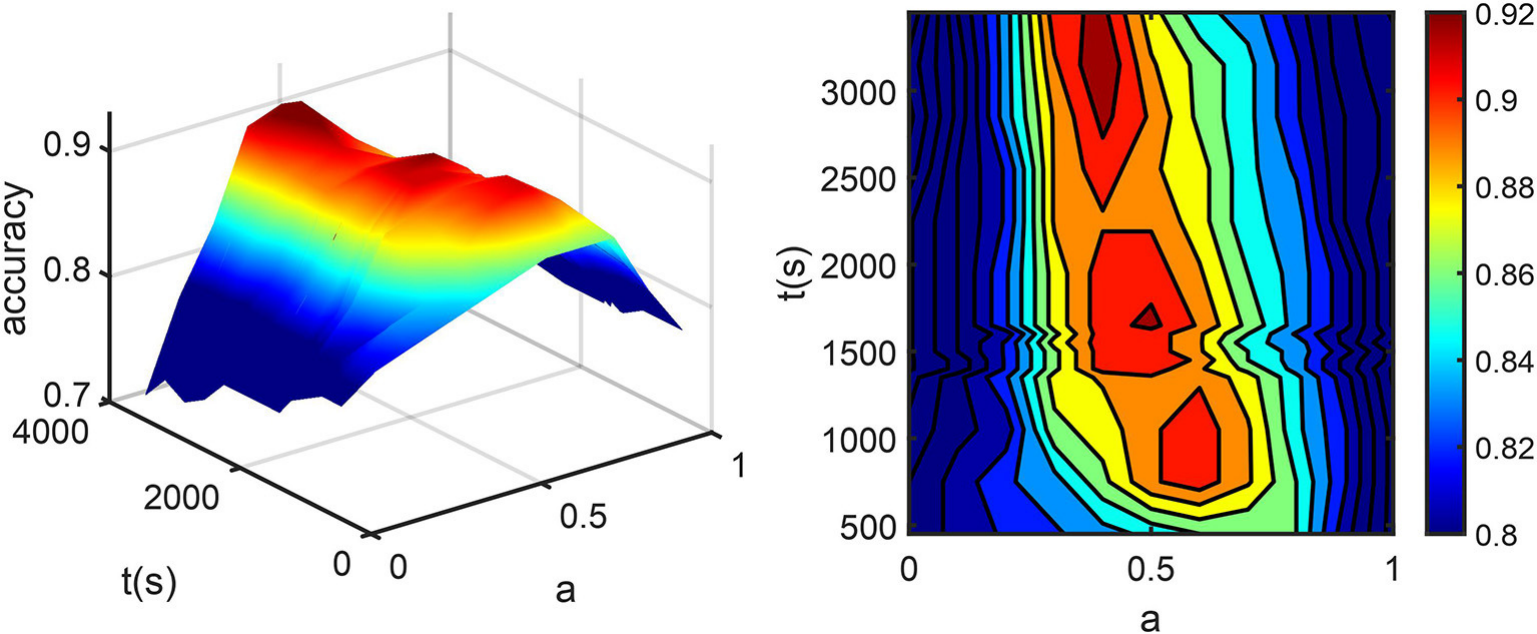
**(A)** The percentage of correctly classified subjects (accuracy) vs. t2 and a. **(B)** accuracy was projected into the a-t plane. Here t1 is fixed at 1,650 s for the convenience of display. The dark red part in the center corresponds to sets of parameters with maximum accuracy, among which the one with minimum scan time was chosen.

Optimal coefficients and time points in Eq. (1) were found by grid search, where the searching intervals were t1 ∈ [0, 3300s], t2∈[300, 3,300 s] with a step size of 50 s and a∈[0, 1] with a step size of 0.1. AQI_roi in the caudal anterior cingulate cortex was then calculated for all training subjects with each set of parameters and used for classifying the AD and HC groups. Figure 3 shows how the parameter selection was performed. The optimal parameters were a = 0.5, t1 = t2 = 1,650 s, resulting in a maximum classification accuracy of 0.92. With these optimized parameters, equation (1) became:


(2) $$\begin{array}{l} \text{AQI\_roi}=0.5\times \frac{S\left(1650\right)-S\left(0\right)}{1650}-0.5\\ \times \frac{S\left(1650\right)-S\left(t_{\max}\right)}{1650-t_{\max}} \end{array}$$

where t_max_ was different for each subject, ranging from 80 to 240 s. Each AQI_roi value was normalized using an injected dose. As a result, one would only need scan data of the first 1,650 s (<30 min). All data after this time point were not necessary for computing AQI.

### Selection of Featured Brain Regions

The contribution of AQI_roi in different brain regions was evaluated using lasso regression. The selected brain regions were caudal anterior cingulate cortex (β = 2.5214) and caudate(β = 0.1976), with the value of coefficient β reflecting their contribution for differentiating AD and HC subjects. An overall AQI was calculated for each subject by linearly combining AQI_roi in the caudal anterior cingulate cortex and caudate:


(3) $$\begin{array}{l} \text{AQ}{\text{I}}_{\text{overall}}=3.6092\times X1+0.2750\times X2+0.5378 \end{array}$$

where *X*1 and *X*2 denote the z-score normalized value of AQI_roi in the caudal anterior cingulate cortex and caudate, respectively. The discriminative accuracy of *AQI*_*overall*_ on the 100 training subjects was 0.93.

### Performance Comparison

#### Performance Evaluation on Training Set

**Figure 4:**
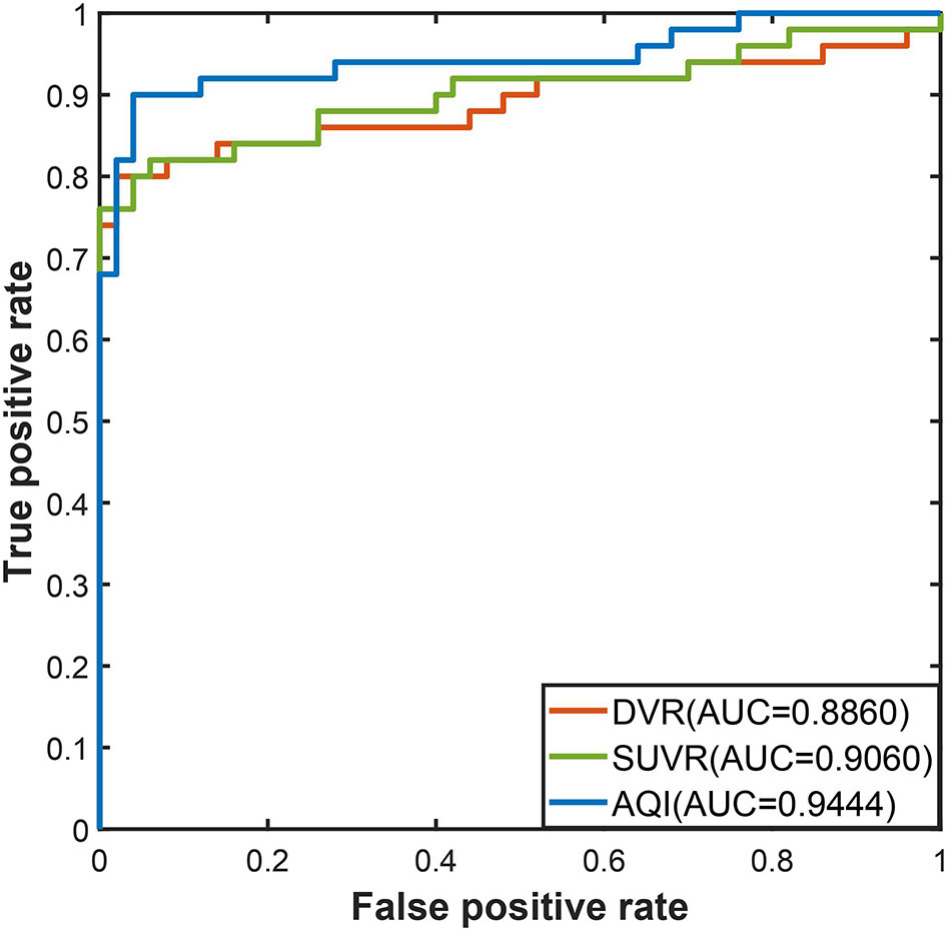
ROC curves for three methods in the training set.

The discriminating performance of SUVR, DVR, and AQI on the 100 training subjects were evaluated and compared using the 10-fold cross-validation. ROC analysis suggested that AQI performed the best in discriminating AD and HC subjects among all three methods. Figure 4 shows the ROC curves for the three methods plotted as the false positive rate against the true positive rate at different classification thresholds. We conclude that AQI performed better than SUVR and DVR, as its curve was above the other two with the highest AUC value of 0.9444. AUC, sensitivity, specificity, accuracy, and optimal threshold for each method are reported in Table 2. All three methods performed well on identifying HC subjects, with the specificity being 0.96 (SUVR), 0.98 (DVR), and 0.96 (AQI) respectively. While SUVR and DVR had an increased error rate for classifying AD subjects, AQI achieved superior performance with a sensitivity of 0.90 over 0.80. The overall accuracy for SUVR, DVR, and AQI was 0.88,0.89, and 0.93, respectively.

**Table 2:** The classification performance of SUVR, DVR, and AQI on training set.

**Methods**	**SUVR**	**DVR**	**AQI**
AUC	0.9060	0.8860	0.9444
sensitivity	0.8000	0.8000	0.9000
specificity	0.9600	0.9800	0.9600
accuracy	0.8800	0.8900	0.9300
optimal threshold	1.4510	1.2795	0.0114

**Figure 5:**
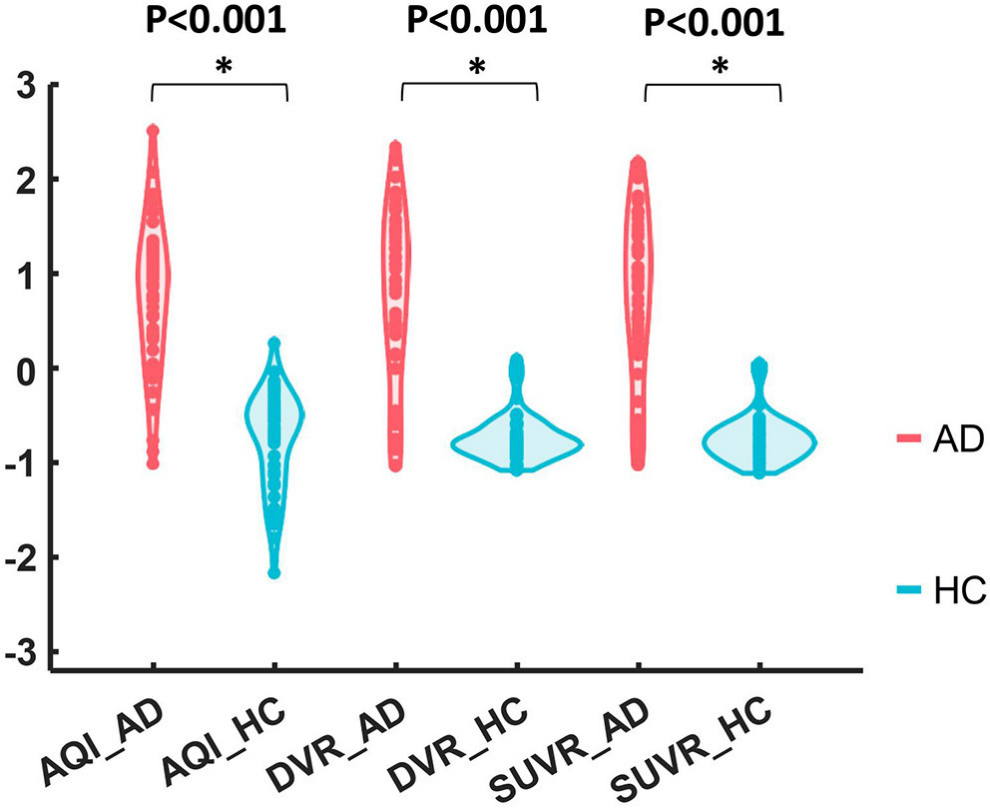
Boxplots of DVR, SUVR, and AQI for AD and HC. For each method, the corresponding data were Z-score normalized to have mean 0 and standard deviation 1 for visual comparison.

Figure 5 shows the boxplots of these three measures for the AD and HC groups. AD subjects had PiB retention in cortical regions and thus had higher values for SUVR and DVR. The median and quantiles of AD were higher than those of HCs for all three measures. AQI measured the difference between PiB retention and the tracer cleaning rate from the brains, which was also more significant in the AD group. The Kruskal–Wallis tests suggested that all three measures could discriminate HC and AD subjects (*p* < 0.001), while AQI had the least degree of overlap on two boxplots. Indeed, Cohen's effect size for SUVR, DVR, and AQI were 2.12, 2.06, and 2.35 respectively, which further proved that AQI was the most effective in discriminating these two groups.

#### Performance Validation on Testing Set

**Figure 6:**
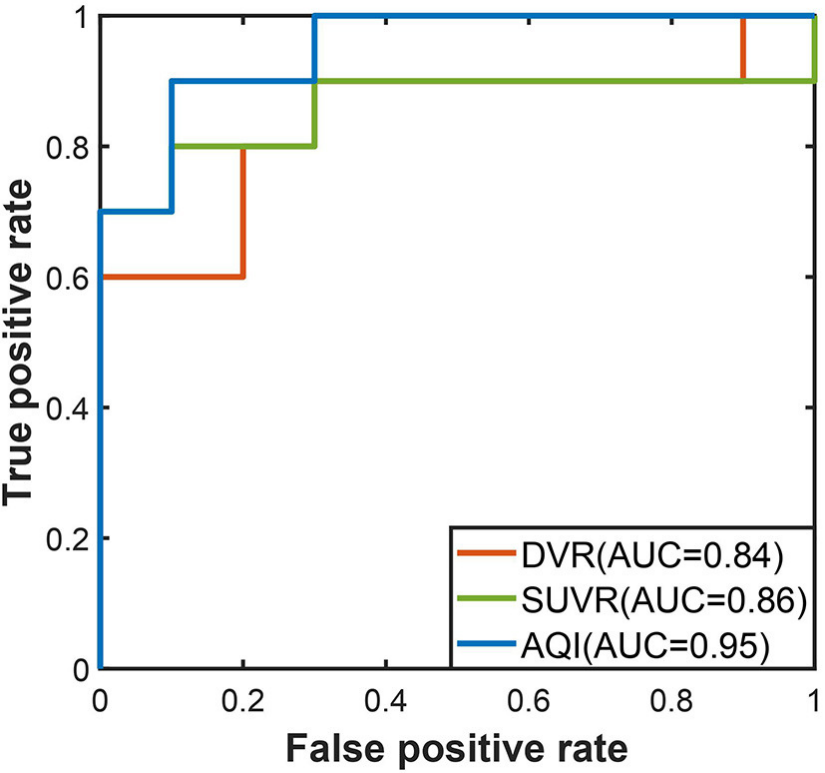
ROC curves for three methods in the testing set.

The performances of the three measures on the testing set were evaluated using the threshold derived from the training dataset. In Figure 6, the ROC curve of AQI was still above those of SUVR and DVR, with the highest value of 0.95. The evaluation metrics in Table 3 indicated that all three methods achieved a sensitivity of 0.8, and that AQI performed better than the other two measures in terms of specificity and overall accuracy. Compared with the training set, all three measures achieved less superior performance on the testing set, although AQI still performed the best among these measures.

**Table 3:** The classification performance of SUVR, DVR, and AQI on testing set.

**Methods**	**SUVR**	**DVR**	**AQI**
AUC	0.8600	0.8400	0.9500
sensitivity	0.8000	0.8000	0.9000
specificity	0.8000	0.8000	0.8000
accuracy	0.8000	0.8000	0.8500
optimal threshold	1.4510	1.2795	0.0114

#### SUVR Images of Selected Samples

**Figure 7:**
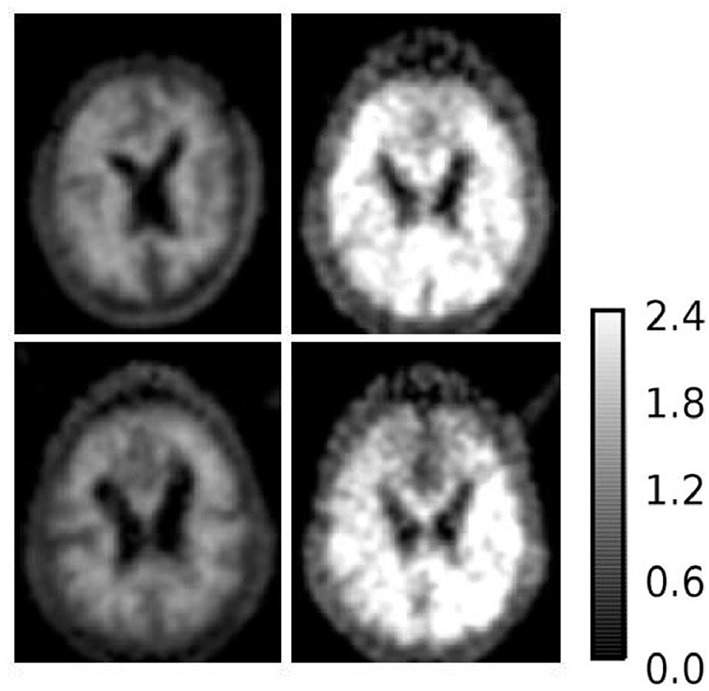
SUVR images of example AD subjects (1) upper left: AD_038 (SUVR = 1.1207); (2) upper right: AD_001 (SUVR = 2.0235); (3) lower left: AD_040 (SUVR = 1.1699); (4) lower right: AD_005 (SUVR = 1.5531). AD_038 and AD_040 were misclassified as HC subjects by SUVR and DVR. AD_001 and AD_005 were correctly classified AD subjects by all three measures.

**Figure 8:**
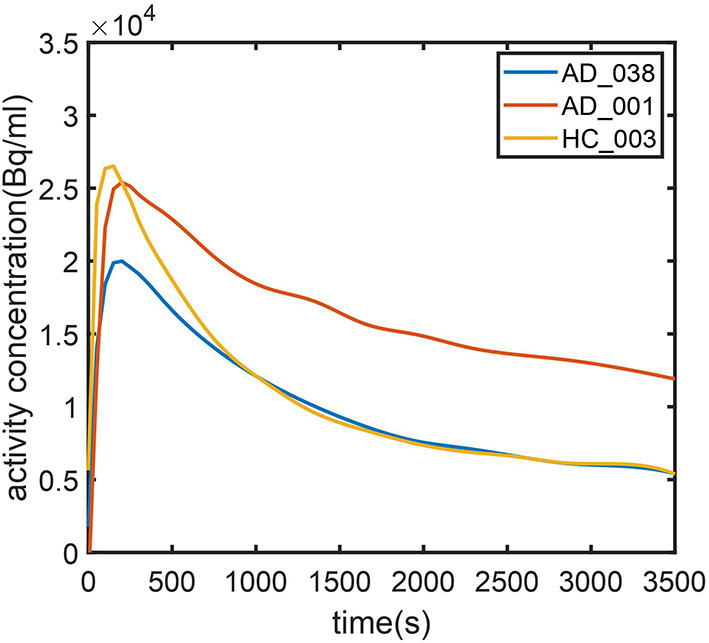
Time–activity curves for (1) typical AD subject (AD_001, blue line) (2) atypical AD subject (AD_038, red line) (3) typical HC subject (HC_003, green line). AD_038 was not identified by SUVR (Figure 6) but was correctly classified by AQI.

To further investigate AD subjects that were misclassified as HC regarding SUVR and DVR, we analyzed SUVR images and TACs of these cases. SUVR images of AD_038, AD_001, AD_040, and AD_005 are shown in Figure 7. All four scans were correctly identified as AD by AQI, while AD_038 and AD_040 were misclassified as HC subjects according to the SUVR and DVR value under the classification threshold. TACs showed that these misclassified AD subjects did not have significant PiB retention or dynamic uptake at the equilibrium stage (see Figure 8), which explained why measures of SUVR and DVR failed to separate them from the HC subjects. This decreased PiB retention is probably due to the lack of fibrillar β-amyloid deposition, as the clearance rate during the clearance period is still more typical of AD subjects (Figure 8). Therefore, by measuring AQI, which considers both retention and clearance rate, these seemingly asymptomatic scans can still be correctly identified.

#### Correlation With MMSE and CDR-SOB Scores

**Figure 9:**
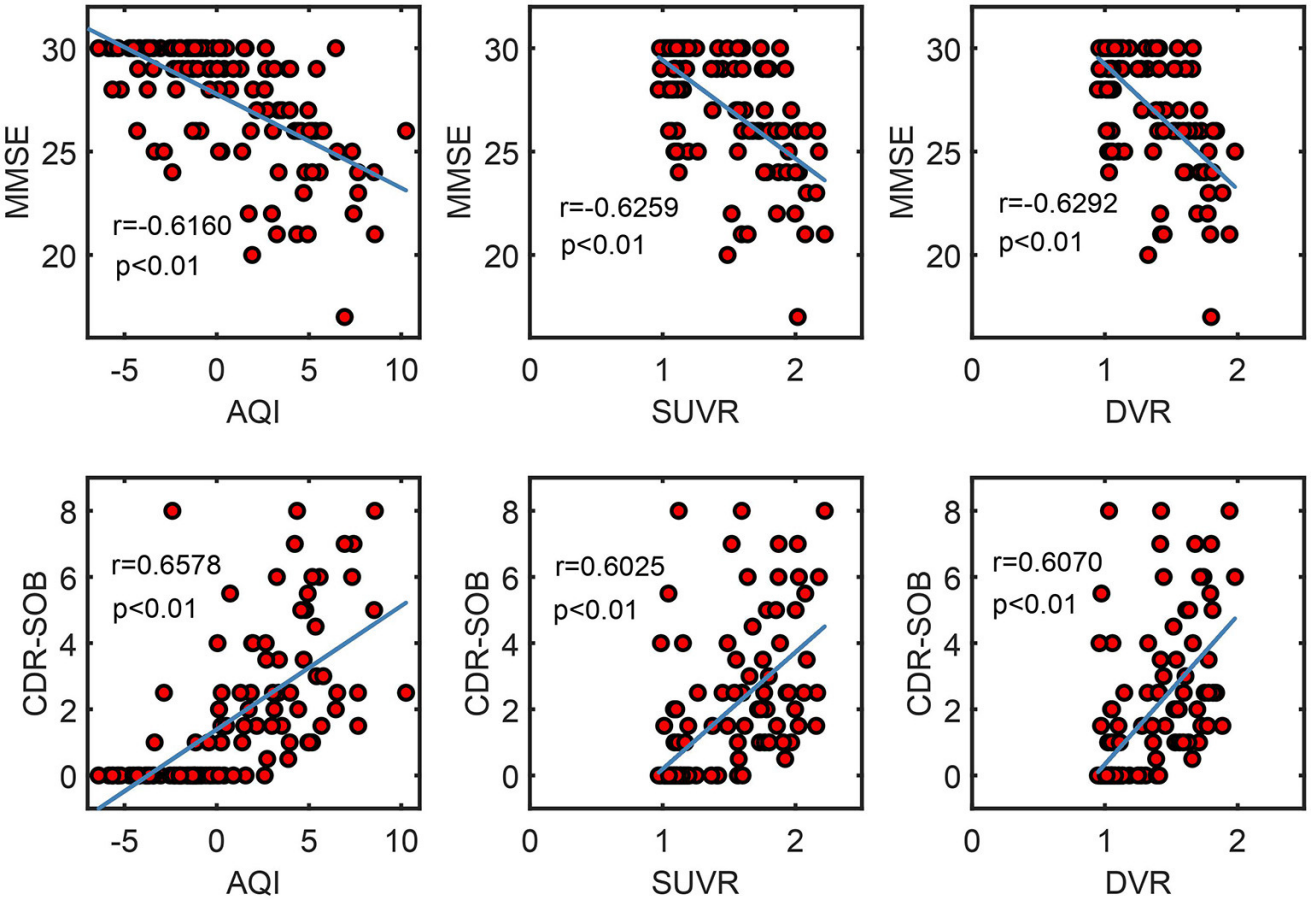
Scatter plots show the correlation between measures (AQI/SUVR/DVR) and clinical scores (MMSE/CDR-SOB). Pearson correlation coefficient r and the corresponding *p*-value were reported for each pair of variables.

Figure 9 shows the correlation between measures and scores of clinical tests (CDR and MMSE). Pearson's correlation coefficients and the significance level were reported for each pair of variables (see Figure 9). For all three measures, their values were proportional to CDR-SOB and inversely proportional to MMSE, with the absolute value of coefficient *r* ranging from 0.60 to 0.66. All of these associations between measures and clinical scores attained statistical significance with *p* < 0.01. AQI did not have a significantly higher correlation with the clinical scores than SUVR/ DVR did.

## Discussion

Amyloid quantification index is a semi-quantitative measure for PiB PET imaging, which is calculated by linearly combining the information corresponding to clearance rate and mid-phase PiB retention. In this study, AQI was shown to effectively distinguish mild AD and HC subjects for 120 scans from a public dataset. AQI achieved an overall accuracy rate of 0.93, which was higher than SUVR and DVR, in the 100 subjects in the training set. The AUC and sensitivity of AQI were also higher than those of SUVR and DVR, while the specificity was comparable. Moreover, the effective size of AQI was 2.35, larger than 2.12 and 2.06, for SUVR and DVR, respectively. Compared with the training set, all three measures achieved less superior performance on the 20 subjects in the testing set, while AQI still performed best among these measures, with the highest accuracy of 0.85.

The AQI can be obtained with the first 30-min scan, which enables a scan protocol with significantly reduced scan/wait time. This could, in turn, improve the scan efficiency, hence reducing the overall cost of a large-scale study. In terms of image quality, a shorter scan has the further advantage of reducing the possibility of motion artifacts (Sureshbabu and Mawlawi, 2005; Dinges et al., 2013). Moreover, the proposed method does not require the selection of a reference region. Using the cerebellar cortex as a reference region could introduce errors as it is not fully devoid of specific binding. A post-mortem study suggested that the widely used reference regions, the cerebellum and the brain stem, were involved in β-amyloidosis when AD progressed into late stages (Thal et al., 2002). Therefore, the SUVR and DVR in target regions could be offset by the increased binding in the reference region.

Unlike SUVR and DVR, which focus on the PiB retention at late scans, AQI accounts for the information of the early-kinetics and mid-phase retention. The underlying concept is in line with several previous studies, which aimed at deriving diagnostic information from early- or mid-stage PiB scans. Blomquist et al. (2008) reported that some patients with AD could not be distinguished regarding PiB retention as they showed equally low PiB uptake ratio in cortical areas as healthy controls, while they still had decreased K1 (influx rate constant), typical of other AD subjects. Therefore, early-phase dynamics can provide extra information when differentiating AD and HC subjects. Sato et al. (2012) showed that the microkinetic parameter k3, estimated from a 28-min scan, could differentiate AD and HC subjects. Jia et al. (2011) reported that the PiB radioactivity clearance rate differed significantly in patients with AD and HCs in the cortex, subcortical nucleus, and pons, with the rate in the AD group being much smaller. Although the actual quantification methods for utilizing early-phase data were different, these researches suggested the importance of exploiting early-phase information.

The AQI combines retention with early kinetics, which enables correctly identifying indistinguishable AD cases regarding PiB retention. For AD_038 and AD_040, which are devoid of enhanced PiB retention in cortical regions, further *in vitro* analysis is needed to confirm whether amyloid deposition is truly absent or is not bound by PiB. One possible explanation is that these subjects are at an early stage or genetically mutated, and thus lack the obvious fibrillar β-amyloid deposition. Previous studies suggested that PiB may be unable to detect AD variants characterized by diffuse β-amyloid plaques as it binds specifically to fibrillar β-amyloid deposition (Bacskai et al., 2007; Ikonomovic et al., 2008). Cairns et al. (2009) reported an ^11^C-PiB-negative AD patient with substantial amounts of diffuse no-fibrillar β-amyloid plaques, as confirmed by the autopsy. Although the PiB scan was performed 2.5 years before the autopsy, the scarcity of fibrillar β-amyloid plaques was unlikely to be identified by PiB–PET imaging even at the time of the autopsy (Cairns et al., 2009). Tomiyama et al. (2008) reported that AD patients with an amyloid precursor protein mutation would have enhanced the formation of β-amyloid oligomers but no fibrilization and displayed very low signal on PiB PET imaging. The above findings suggested that PiB retention was not completely reliable for AD identification as it may overlook certain AD cases. In our study, the false negative AD cases still display symbolic pathological changes in terms of PiB dynamics and thus can be correctly identified using AQI (Figure 8). Moreover, one HC subject without significant PiB retention was misclassified as AD by AQI. This subject was later diagnosed as having uncertain dementia, which suggested that AQI may have detected early-stage symptoms of AD based on abnormal PiB kinetics. Another possibility would be that the ground truth used in this study may be inaccurate as even clinical AD diagnosis can be inaccurate since AD can only be definitely diagnosed neuropathologically at autopsy. If this is true, some of the correctly classified AD subjects, e.g., Figure 7, can actually be because of cognitive impairment due to non-AD causes. Whether this is valid or not is subject to further neuropathological support.

There are several limitations of this study. One limitation is that the current results were based on subjects from a single source of dataset and thus may not apply to others. One conclusion of this article is that by exploiting both clearance rate and PiB retention, the performance of differentiating mild AD and HC subjects is superior to using PiB retention alone, while the actual performance may vary across datasets acquired at different centers with various models of scanners. Although, it can be difficult to obtain full dynamic scans to test the proposed method, as most of the centers currently execute a late-scan protocol. A second limitation is that during subject selection those patients with non-AD dementia were excluded, while the clinical situation can be more complicated as diseases such as frontotemporal dementia and Lewy body dementia are likely to interfere with the diagnosis of AD. Future work is needed to test whether AQI will be affected by other types of dementia. The third limitation is that a more appropriate normalization method requires to be investigated as we found that normalization simply by dose only achieved comparable results to ones even without normalization. The last limitation is that the logistic regression classifier used for classifying subjects in this study is probably not the best choice. Other machine learning techniques, e.g., support vector machines, could be used to further improve the performance. However, the main goal of this work is to propose and validate a measure with short scan time and acceptable accuracy in differentiating mild AD and HC subjects.

In the future, AQI can be tested on differentiating MCI from AD and HC and predicting MCI progression. AQI can be applied in combination with MRI imaging, which may provide stronger evidence and achieve greater accuracy than using either of the imaging modality alone (Patel et al., 2020). Another possibility is to explore whether AQI can be applied to PET data obtained with other amyloid imaging agents, such as ^18^F-Florbetapir. It is expected ^18^F-Florbetapir and PiB share similar kinetics, which could enable AQI to simplify the ^18^F-Florbetapir scan protocol.

## Data Availability Statement

Data were provided by OASIS-3 dataset, which is publicly available via the website: https://www.oasis-brains.org/ (principal investigators: T. Benzinger, D. Marcus, and J. Morris; NIH P50AG00561, P30NS09857781, P01AG026276, P01AG003991, R01AG043434, UL1TR000448, and R01EB009352).

## Ethics Statement

The studies involving human participants were reviewed and approved by Washington University School of Medicine. The patients/participants provided their written informed consent to participate in this study.

## Author Contributions

TS and CS: conception and design. TS, YY, DL, XL, HZ, and MW: administrative support. CS and TS: provision of study materials or patients. CS, TS, ZW, and HC: collection and assembly of data. TS, YY, HW, ZW, HC, YB, and XL: data analysis and interpretation. All authors: manuscript writing and final approval of manuscript.

## Funding

This work is supported by the Scientific Instrument Innovation Team of the Chinese Academy of Sciences (GJJSTD20180002), the Key Laboratory for Magnetic Resonance and Multimodality Imaging of Guangdong Province (2020B1212060051), the Chinese Academy of Sciences Engineering Laboratory for Medical Imaging Technology and Equipment (KFJ-PTXM-012), the National Natural Science Foundation of China (Nos. 81729003 and 61871373), the Pearl River Talent Recruitment Program of Guangdong Province (No. 2019QN01Y986), the Shenzhen Science and Technology Program (JCYJ20210324115810030), and the Shenzhen Peacock Plan Team Program (KQTD20180413181834876).

## Conflict of Interest

The authors declare that the research was conducted in the absence of any commercial or financial relationships that could be construed as a potential conflict of interest.

## Publisher's Note

All claims expressed in this article are solely those of the authors and do not necessarily represent those of their affiliated organizations, or those of the publisher, the editors and the reviewers. Any product that may be evaluated in this article, or claim that may be made by its manufacturer, is not guaranteed or endorsed by the publisher.
